# Emerging infectious disease issues in blood safety.

**DOI:** 10.3201/eid0707.010731

**Published:** 2001

**Authors:** M E Chamberland, H J Alter, M P Busch, G Nemo, M Ricketts

**Affiliations:** Centers for Disease Control and Prevention, Atlanta, Georgia 30333, USA. mchamberland@cdc.gov

## Abstract

Improvements in donor screening and testing and viral inactivation of plasma derivatives together have resulted in substantial declines in transfusion-transmitted infections over the last two decades. Most recently, nucleic acid testing techniques have been developed to screen blood and plasma donations for evidence of very recent viral infections that could be missed by conventional serologic tests. Nonetheless, the blood supply remains vulnerable to new and reemerging infections. In recent years, numerous infectious agents found worldwide have been identified as potential threats to the blood supply. Several newly discovered hepatitis viruses and agents of transmissible spongiform encephalopathies present unique challenges in assessing possible risks they may pose to the safety of blood and plasma products.


### Emerging Infectious Disease Issues in Blood Safety

**Mary E. Chamberland,* Harvey J. Alter,† Michael P. Busch,‡ George Nemo,† and Maura Ricketts§**

*Centers for Disease Control and Prevention, Atlanta, Georgia, USA; †National Institutes of Health, Bethesda, Maryland, USA; ‡Blood Centers of the Pacific, San Francisco, California, USA; §World Health Organization, Geneva, Switzerland

Improvements in donor screening and testing and viral inactivation of plasma derivatives together have resulted in substantial declines in transfusion-transmitted infections over the last two decades. Most recently, nucleic acid testing techniques have been developed to screen blood and plasma donations for evidence of very recent viral infections that could be missed by conventional serologic tests. Nonetheless, the blood supply remains vulnerable to new and reemerging infections. In recent years, numerous infectious agents found worldwide have been identified as potential threats to the blood supply. Several newly discovered hepatitis viruses and agents of transmissible spongiform encephalopathies present unique challenges in assessing possible risks they may pose to the safety of blood and plasma products.

### Nucleic Acid Testing

The risk of transfusion-transmitted viral infections is primarily due to the failure of serologic screening tests to detect recently infected donors in the preseroconversion "window" phase of infection. To reduce this window period, European Union regulators began to require in 1999 that all plasma be tested by nucleic acid testing (NAT) techniques for hepatitis C virus (HCV) if derivatives made from such plasma were to be sold in Europe. This announcement was a major impetus for developing and implementing NAT of blood and plasma from donors in the United States and other developed countries.

Virtually all whole blood and plasma donations collected in the United States are being screened for both HCV and HIV-1 by NAT. The testing is being done as a part of investigational new drug applications approved by the U.S. Food and Drug Administration. Due to the complex and labor-intensive nature of the testing, it is being implemented by using a pooled strategy-–namely, donations are being tested in pools of 16 to 24. At this nascent stage, NAT can require several days more to complete than conventional serologic tests. Certain components, particularly platelets because they become outdated in 5 days, are being released by some blood centers before NAT has been completed and on the basis of serologic testing alone.

The pooled NAT procedure is expected to reduce the preantibody seroconversion window period from the current 22 days to about 12 days for HIV and from 70 days to 10 to 14 days for HCV. Organisms are potentially detectable only during a portion of the pre-NAT window period, even when using single donor NAT. It is this portion of the window period when donations are thought to be viremic (i.e., infectious) that is critical to transfusion safety.

Address for correspondence: Mary E. Chamberland, Centers for Disease Control and Prevention, 1600 Clifton Road, Mailstop A30, Atlanta, GA 30333, USA; fax: 404-639-3163; e-mail: mchamberland@cdc.gov

Testing of whole blood donations in the United States from April 1999 through March 2000 revealed 42 NAT-positive/HCV antibody-negative donations (of 1.04 x $10^7$ tested) and 4 NAT-positive/HIV-1 antibody-negative/HIV-1 p24 antigen-negative donations (of 0.76 x $10^7$ tested). The rate of HCV-infected donations positive by only NAT was 40.3 (approximately 1 in 250,000) and HIV-infected was 5.2 (approximately 1 in 2,000,000) per 10 million donations. These testing advances, however, are associated with substantial costs: the mini-pool NAT procedure has been estimated to cost $1.2 million dollars per quality-adjusted life year.

As the technology evolves, NAT testing of all blood components will be completed prior to transfusion. The ultimate goal is to progress from mini-pool testing to single donor testing, but this change appears to be several years away. Future applications of NAT technology may include expanding testing platforms to enable direct detection of additional viruses (e.g., hepatitis B virus, parvovirus B-19, and cytomegalovirus) and other infectious agents (e.g. *Trypanosoma*, *Babesia*, and *Plasmodium* species).

### Novel Hepatitis Agents

Although important advances have been made in our understanding of transfusion-transmitted hepatitis over the last several decades, some persons with acute posttransfusion hepatitis test negative for all known hepatitis agents. This observation has fueled concerns about the existence of one or more as-yet-unidentified hepatitis viruses that can be transmitted by blood transfusion. Through advances in molecular virology several "candidate" viruses have been identified as the cause of non-A-E hepatitis.

TT virus (TTV), named for the patient from whom it was first isolated in Japan, is a novel, single-stranded, circular DNA virus. Although TTV can be transmitted by transfusion, similar rates of infection have been observed among blood recipients tested posttransfusion who did and did not develop transfusion-associated hepatitis. TTV can result in persistent infection; however, studies to date have not shown an associated pathologic condition, and no current evidence suggests that TTV is an agent of hepatitis in humans.

Recently, much attention has been focused on a diverse family of viruses called SEN-V that were isolated by using degenerate primers from TTV. To date, preliminary, limited studies have found that approximately 2% of current and pre-1990 blood donors test positive for SEN-V. Testing of archived serum samples at the National Institutes of Health (NIH) showed that the proportion of cardiac surgery patients with evidence of new infection with SEN-V was 10 times higher among those who had received blood transfusions (30%) than among those who had not (3%). Further, a SEN-V-positive donor could be identified for about 70% of SEN-V-positive recipients. Although these data clearly indicate that SEN-V is transmitted by transfusion, many important questions remain unanswered; for example, is SEN-V the long-anticipated primary agent of non-A-E hepatitis? The NIH study found new SEN-V infections in 11 of 12 patients (92%) with non-A-E hepatitis and 60 of 252 patients (24%) who did not develop hepatitis ($p<0.001$). In addition, the level of viremia generally paralleled the alanine aminotransferase level. These data suggest, but do not prove, a causal association with transfusion-transmitted non A-E hepatitis. Early evidence suggests that SEN-V can replicate in the liver, but there are no data to show that it is a cause of fulminant liver failure, and its role in cirrhosis and chronic cryptogenic hepatitis is uncertain.

### Transmissible Spongiform Encephalopathies

Creutzfeldt-Jakob disease (CJD) is a human transmissible spongiform encephalopathy believed to be caused by an unconventional agent—a prion protein—which is an altered form of a normal protein found in many tissues of the body. Most cases of CJD are classified as sporadic, since they are thought to result from spontaneous generation of the abnormal prion protein, which then continues to replicate and accumulate in the brain. Between 10% and 15% of persons with CJD have familial disease, caused by one of more than 20 known mutations. A small number (250) of iatrogenic cases of CJD have occurred in persons who received contaminated pituitary growth hormone, corneas, or dura mater from human cadavers or who were operated on with contaminated neurosurgical instruments.

Concerns regarding the transmissibility of the agent of CJD by blood are supported primarily by laboratory and experimental studies. These studies have demonstrated that rodents with several experimental transmissible spongiform encephalopathies (TSE) have small amounts of infectivity in blood during both the asymptomatic incubation period and clinically overt disease. Transfusion transmission of an experimental TSE in hamsters has been demonstrated, albeit rarely, when known infected blood was administered intravenously.

Despite the findings that suggest a potential for bloodborne transmission of CJD, accumulating epidemiologic data support the view that such a risk, if it exists at all, remains theoretical. First, there are no confirmed reports of CJD transmission by blood or blood products, despite intensive efforts to identify such cases. Second, five case-control studies involving nearly 2,500 patients have not shown blood transfusions to be a risk factor for CJD. Third, CJD has not been detected in recipients of blood from donors who developed CJD months to years after donating blood and were presumably incubating CJD at the time of donation. Finally, active surveillance was conducted among an estimated 12,000 hemophilia patients in hemophilia treatment centers in the United States since 1995, plus additional centers in other countries, and no cases of CJD have been found among them.

In 1996, a new variant of CJD (nvCJD) was first recognized in the United Kingdom. The cause of this new human transmissible spongiform encephalopathy appears to be the same agent responsible for an outbreak of bovine spongiform encephalopathy (BSE) among cattle in the United Kingdom. The BSE epizootic is thought to have resulted from inclusion of carcasses of sheep infected with scrapie in the meat and bone meal fed to cattle in the early 1980s. Features of nvCJD are distinctly different from those of sporadic CJD; for example, patients infected with nvCJD are younger and have prominent early psychiatric and behavioral manifestations, and nvCJD has a distinctive neuropathology. As of July 2000, public health officials had reported 75 cases of confirmed or probable nvCJD in the United Kingdom; 2 cases were found in France and 1 in Ireland.

No cases of transmission of nvCJD by blood transfusion have been reported; nonetheless, because its agent is newly discovered, its degree of infectiousness is unknown. Further, important differences have been noted between sporadic CJD and nvCJD, and it is therefore impossible to extrapolate about nvCJD based on what is known about sporadic CJD. For example, the abnormal prion protein is detectable in tissues taken from spleens and tonsils of patients with nvCJD but not in those taken from patients with sporadic CJD. In view of this uncertainty, U.K. health officials have taken several precautions, which include retrieving all blood and blood products made with blood or plasma obtained from donors subsequently identified with nvCJD, importing all plasma used to produce plasma-derived products, and implementing leukofiltration of all blood donations. Persons who resided in or traveled to the United Kingdom for a total of 6 months or more between 1980 and 1996 are not permitted to donate blood and plasma in the United States.

### Conclusion

The high safety level of the blood supply is the result of continued refinements and improvements in donor screening and testing. Continued vigilance is critical to protect the blood supply from known pathogens and to detect the emergence of new infectious agents. As potential new threats are discovered, the need for both the safety of the blood supply and the availability of lifesaving blood and blood products must be balanced. Finally, because the margin of benefit is likely to be very small in some cases, especially when the risk of transmitting some infectious agents via transfusion is very low, policymakers may need to consider economic factors as well as health factors when making decisions involving the blood supply.

